# Analysis of surgically induced astigmatism of the anterior, posterior, and total cornea after implantable collamer lens implantation: a comparative study between temporal and superior clear corneal incisions

**DOI:** 10.1186/s12886-024-03501-x

**Published:** 2024-06-12

**Authors:** Ting Wan, Huaicheng Chen, Shirou Wu, Hongying Jin

**Affiliations:** 1 Eye Center, Second Affiliated Hospital, School of Medicine, Zhejiang University, 88 Jiefang Road, Hangzhou, 310009 China; 2 Department of Ophthalmology, The Fourth Affiliated Hospital, Zhejiang University School of Medicine, Yiwu, Zhejiang China

**Keywords:** Surgically induced astigmatism, Cornea, Incision site, Implantable collamer lens

## Abstract

**Background:**

To comparatively analyze the surgically induced astigmatism (SIA) of the anterior, posterior, and total corneas of eyes undertaking implantable collamer lens (ICL) implantation with temporal or superior corneal incisions.

**Methods:**

One hundred and nine eyes of 109 patients who received ICL implantation were recruited: 40 eyes had temporal incisions and 69 eyes had superior incisions. Total corneal refractive power (TCRP); simulated keratometry of the anterior (Sim-K_Ant_) and posterior (Sim-K_Post_) corneal curvature; and astigmatism of the anterior (CA_Ant_), posterior (CA_Post_), and total (CA_TCRP_) cornea were recorded through a Pentacam preoperatively and 3 months postoperatively. The SIA of the anterior, posterior, and total cornea were also compared between the two groups.

**Results:**

There were no significant intergroup differences for TCRP, Sim-K_Ant_, Sim-K_Post_, CA_Ant_, CA_Post_, or CA_TCRP_, preoperatively. However, values of CA_Ant_, CA_Post_, and CA_TCRP_ with temporal incision were significantly higher than those parameters with superior incision postoperatively. All of the SIA of the anterior, posterior, and total cornea were significantly lower for temporal incision than those with a superior incision (*p* < 0.001, *p* = 0.006 and *p* = 0.001 respectively). Meanwhile, the superior incisions created against-the-rule (ATR) astigmatism, and temporal incisions always induce with-the-rule (WTR) astigmatism in total cornea.

**Conclusions:**

A superior incision may be suitable for correcting WTR astigmatism, while a temporal incision for correcting ATR astigmatism when using a non-toric ICL. Meanwhile, temporal incision could be a better choice with little preoperative astigmatism or that preoperative astigmatism would be corrected with toric ICLs.

**Trial registration:**

Registration number: ChiCTR2100051739. Prospectively registered: 01 October 2021.

## Background

The EVO Visian Implantable Collamer Lens (ICL) is widely accepted as an effective and secure procedure for correcting myopia and astigmatism [1–8]. For an increasing number of patients choosing the ICL procedure, more effort should be paid to minimizing both postoperative spherical and cylindrical refraction for the best visual outcome. Considering that ICL implantation requires the creation of a clear corneal incision of 3.0 mm, corneal surgically induced astigmatism (SIA) is a common problem in almost all the eyes that undertake this corneal incision, and it remains one of the most common complications. However, the ICL power is presently determined using the calculator provided on the ICL manufacturer’s website, which overlooks the role that SIA plays in the refractive results when evaluating the ICL astigmatic power [1]. Thus, an exact evaluation of corneal curvature before and after surgery is required to explore the incisions induced corneal astigmatism.

Until now, few reports have explored the change in shape and SIA of the cornea after ICL implantation. Furthermore, the studies acquired only the anterior corneal curvature to evaluate corneal astigmatism by using the keratometer or corneal topography, as it was previously difficult to measure the posterior corneal surface previously [9, 10]. However, corneal astigmatism is determined by the anterior as well as the posterior corneal surface. Even though the keratometric index was developed to compensate those measurements with only the anterior curvature of the cornea, significant error still exists when estimating the actual corneal power [11]. Thus, besides the anterior corneal surface, it is important to evaluate the change of the cornea posterior surface to fully understand corneal incision-induced shape change and astigmatism. With the development of the Pentacam, a diagnostic device using a slit illumination system and modeling on the Scheimpfug principle, the posterior surface of the cornea can be directly measured in addition to the anterior surface [12]. To the best of our knowledge, the posterior and total shape change of the cornea after ICL implantation has not yet been studied.

Thus, we conducted this study to comparatively evaluate the refractory power and astigmatism of the anterior, posterior, and total cornea after ICL implantation with temporal and superior incisions. The SIA of the anterior, posterior, and total cornea were also compared between the two groups. In this study, SIA was evaluated by vector analysis so that both the magnitude and axis of corneal astigmatism change could be shown. This study provides valuable information about the incision-induced cornea shape change and astigmatism associated with different incision sites (i.e., posterior and total cornea), in addition to the anterior surface, which will help us choose a more suitable incision site.

## Methods

This is a prospective study recruited patients who undertook implantable collamer lens implantation with temporal (Group T) or superior (Group S) corneal incisions from Oct 2021 to May 2022. The right eye of each patient was chosen for analysis. The incision site was mainly determined based on both the magnitude and direction of the preoperative cornea astigmatism and whether the preoperative astigmatism would be corrected with toric ICLs. The orientation of astigmatism were classified based on Sim-K_ant_ of Pentacam. For eyes with with-the-rule (WTR) corneal astigmatism more than 0.75 D and intended to be implanted with non-toric ICL, superior corneal incisions would be chosen. For the other eyes, temporal corneal incisions would be chosen. Finally, one hundred and nine eyes of 109 patients (40 cases in Group T and 69 cases in Group S) finished the 3 months follow up. All surgeries were carried out by one experienced surgeon (JHY). This prospective study adhered to the tenets of the Declaration of Helsinki and was approved by the Institutional Review Board of the Ethics Committee of Second Affiliated Hospital, School of Medicine, Zhejiang University. All the patients recruited in the study signed the informed consent form before the commencement of the study. The study has been registered in http://www.chictr.org.cn as No.ChiCTR2100051739.

### Surgical technique

The ICL power was calculated using the calculation tool provided by the ICL manufacturer (STAAR Surgical Company) on its website. The ICL sizes were determined depending on the corneal horizontal white to white distance, which was measured using the Pentacam device (Oculus, Wetzlar, Germany). All the surgeries were carried out in accordance with standard procedures as previously described [13, 14]. The patients were given tropicamide phenylephrine eye drops (Santen, Osaka, Japan) for pupil dilating and Alcaine (0.5% proparacaine hydrochloride) Eye Drops (Alcon, Fort Worth, TX, USA) for surface anesthesia preoperatively. After making a 3.0 mm clear corneal incision, a loaded V4C ICL was inserted into the anterior chamber. Subsequently, the viscoelastic agent was injected to maintain the depth of the anterior chamber, followed by ICL being placed into the posterior chamber and rotated to the predesigned axis. Finally, the remaining viscosurgical agent in the eye was washed out completely. After surgery, tobramycin and dexamethasone eye drops (S.A. Alcon-Couvreur, N.V.) was topically administered four times daily for 1 week.

### Evaluation of corneal shape and SIA

Routine ophthalmic examinations were performed before and 3 months after surgery. Parameters of corneal shape including total corneal refractive power (TCRP); simulated keratometry of the anterior (Sim-K_Ant_) and posterior (Sim-K_Post_) corneal curvature; and astigmatism of the anterior (CA_Ant_), posterior (CA_Post_), and total (CA_TCRP_) cornea were recorded through a Placido–Scheimpflug system, Pentacam (Oculus, Germany) [15–17] preoperatively and 3 months postoperatively. To assess quality, the specification of the measurements should be “OK,” so they can be accepted for later analysis. The data of the central 3.0 mm zone were used for analyzing and comparison. Moreover, central corneal thickness and peripheral corneal thicknesses in the superior and temporal quadrants were obtained from the Pentacam.

SIA was determined as the change vector from the preoperative astigmatism to postoperative astigmatism. In this study, vector analysis was used to calculate SIA. The astigmatism double angle plot tool provided on the American Society of Cataract and Refractive Surgery website (https://ascrs.org/tools/corneal-sia-tool) was used to display the double angle plots for corneal SIA distributions [18] .

### Statistical analysis

SPSS software (version 22; SPSS, Chicago, IL, USA) was used to conduct the statistical analyses. The normality of all data samples was tested using the Kolmogorov–Smirnov test. Comparisons between Group T and Group S were analyzed using an unpaired t-test for data with a normal distribution, and the Mann–Whitney U test was used for data with a non-normal distribution, both with Bonferroni correction. Comparisons between preoperative and postoperative values were performed using a paired sample t-test for data with a normal distribution, and Wilcoxon’s test was used for data with a non-normal distribution, both with Bonferroni correction. Comparisons among the SIA of total, anterior and posterior cornea in each incision group were performed using Analysis of Variance with Bonferroni test.

## Results

### Study population

Table 1 illustrates the preoperative demographic characteristics of the eyes of the patients recruited for the study. With Bonferroni correction, a p value of < 0.0045 was considered statistically significant. No significant differences were found for age, spherical equivalent, sphere refraction, cylinder refraction, intraocular pressure, endothelium cell density (ECD), corneal thickness, or ICL power. However, the incision corneal thickness was larger in Group S as compared to the values in Group T patients (*p* < 0.001).

**Table 1:** Preoperative demographic information of eyes undergoing ICL implantation (mean ± SD and range)

Parameter	Group T	Group S	*p*
Eye(n)	40	69	
Male/Female(n)	8/32	12/57	
Age(y)	25 ± 4.7(17 to 38)	26 ± 4.9(18 to 42)	0.35
SE(D)	-9.84 ± 2.83(-18.5 to-3.75)	-9.37 ± 2.95(-21.5 to -3.5)	0.42
Sphere (D)	-9.24 ± 2.74(-17.75 to-3.25)	-8.93 ± 2.84(-20 to -3.5)	0.58
Cylinder (D)	-1.21 ± 1.09(-3.75 to 0)	-0.89 ± 0.67(-3 to 0)	0.06
IOP(mmHg)	16 ± 3.0(9 to 22)	15.7 ± 2.9(10 to 23)	0.63
ECD(cell/mm^2^)	2800 ± 241(2380 to 3210)	2720 ± 215(2274 to 3344)	0.09
CCT(µm)	527 ± 32(463 to 591)	521 ± 34(457 to 620)	0.33
PCT(temporal) (µm)	601 ± 38(528 to 671)	594 ± 35(521 to 683)	0.31
PCT(Superior) (µm)	658 ± 38(578 to 761)	652 ± 39(576 to 751)	0.46
IOL power (D)	-9.29 ± 2.77(-17.75 to -3.25)	-9.14 ± 2.70(-18 to -3.5)	0.82
Incision thickness(µm)	601 ± 38(528 to 671)	652 ± 39(576 to 751)	< 0.001*
Ratio of WTR/ATR/Oblique(%)	92.5/2.5/5	100/0/0	

Group T: Temporal incision; Group S: Group with superior incision; SD: standard deviation; D: diopters; SE: Spherical Equivalent; IOP: intraocular pressure; ECD: endothelium cell density; CCT: central corneal thickness; PCT: peripheral corneal thickness; IOL: implantable collamer lens; y = year; WTR: with-the rule; ATR: against-the-rule

With Bonferroni correction, a p value of < 0.0045 was considered statistically significant

* Significant difference between Group T and Group S

The keratometry values preoperatively and 3 months postoperatively, as stratified by site of clear corneal incision, are shown in Table 2. With Bonferroni correction, a p value of < 0.008 was considered statistically significant. The results show no significant differences between the two preoperative groups for TCRP (*p* = 0.061), anterior simulated keratometry (Sim-K_Ant_) (*p* = 0.084), posterior simulated keratometry (Sim-K_Post_) (*p* = 0.21), anterior corneal astigmatism (CA_Ant_) (*p* = 0.775), posterior corneal astigmatism (CA_Post_) (*p* = 0.712) or total corneal astigmatism (CA_TCRP_) (*p* = 0.626).

**Table 2:** Comparison of keratometry values before and 3 months after ICL implantation as stratified by site of clear corneal incision (mean ± SD)

Incision	Temporal		Superior	
Time	Pre-op	Post-op 3 m	Pre-op	Post-op 3 m
Sim-K_Ant_ (D)	43.42 ± 1.33	43.44 ± 1.28	43.85 ± 1.17	43.83 ± 1.20
CA_ant_ (D)	1.41 ± 1.02^#^	1.69 ± 0.96*^#^	1.36 ± 0.63^#^	0.88 ± 0.60*^#^
Sim-K_post_ (D)	-6.33 ± 0.20	-6.33 ± 0.20	-6.38 ± 0.21	-6.37 ± 0.19
CA_post_ (D)	-0.38 ± 0.18^#^	-0.45 ± 0.17*^#^	-0.39 ± 0.12^#^	-0.27 ± 0.13*^#^
TCRP K_mean_ (D)	42.71 ± 1.30	42.71 ± 1.39	43.19 ± 1.26	43.12 ± 1.30
CA_TCRP_ (D)	1.38 ± 1.07^#^	1.64 ± 1.00*^#^	1.29 ± 0.65^#^	0.87 ± 0.62*^#^

SD: standard deviation; D: diopters; Sim-K_Ant_: anterior simulated keratometry; CA_ant_; anterior corneal curvature astigmatism; Sim-K_Post_: posterior simulated keratometry; CA_post_: posterior corneal curvature astigmatism; TCRP: total corneal refractive power ; CA_TCRP_: total corneal refractive power astigmatism

With Bonferroni correction, a p value of < 0.008 was considered statistically significant

^*^Significant difference of postoperative value between Group T and Group S

^#^ Significant difference between preoperative and 3 months postoperative value in each group

**Fig. 1:**
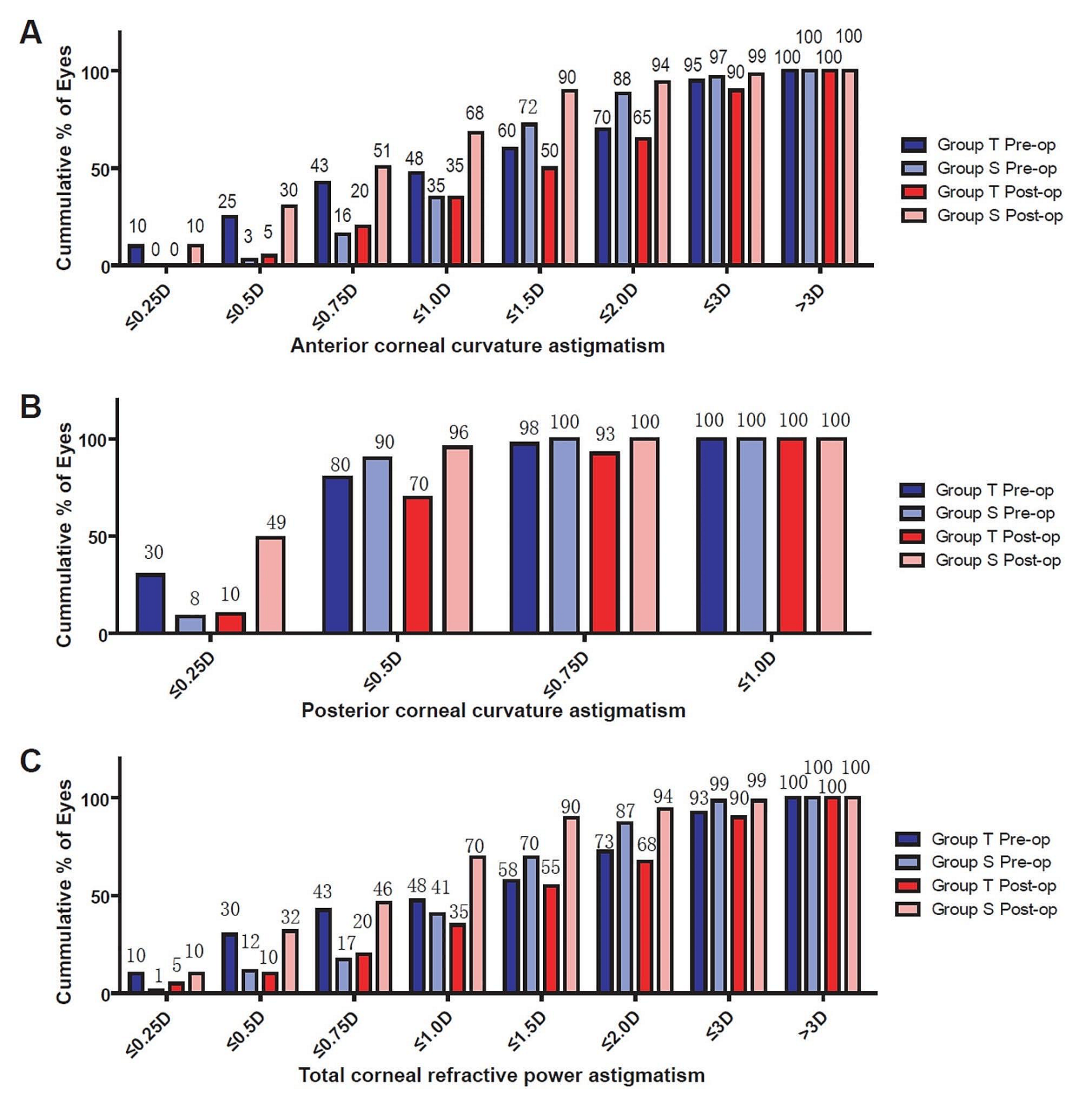
Cumulative frequency of CAAnt (**A**), CAPost (**B**), and CATCRP (**C**) preoperatively and at 3 months postoperatively. CAAnt: anterior corneal curvature astigmatism; CAPost: posterior corneal curvature astigmatism; CATCRP: total corneal refractive power astigmatism

Three months postoperatively, no significant difference was found between Group T and Group S for TCRP (*p* = 0.126), Sim-K_Ant_ (*p* = 0.106), or Sim-K_Post_ (*p* = 0.260). Moreover, no significant differences were found between pre- and post-operative values for TCRP, Sim-K_Ant_, and Sim-K_Post_ in either Group T or Group S (*p* = 0.957, *p* = 0.706, *p* = 0.785, respectively in Group T, and *p* = 0.237, *p* = 0.625, *p* = 0.605, respectively in Group S). However, CA_Ant_, CA_Post_, and CA_TCRP_ values significantly increased in Group T (all *p* < 0.001), while those values significantly decreased in Group S 3 months postoperatively (all *p* < 0.001). As a result, CA_Ant_, CA_Post_, and CA_TCRP_ values in Group T were significantly higher than those values in Group S 3 months postoperatively(all *p* < 0.001). The cumulative frequencies of CA_Ant_ (A), CA_post_ (B), and CA_TCRP_ (C) before and 3 months after surgery are presented in Fig. 1.

Meanwhile, SIA of anterior, posterior, and total cornea with temporal and superior corneal incisions are presented in Table 3, which shows that all of the SIA of anterior (SIA_Ant_), posterior (SIA_Post_), and total cornea (SIA_TCRP_) in Group T was significantly lower as compared to those values in Group S (*p* < 0.001 for SIA_Ant_, *p* = 0.006 for SIA_Post_, *p* = 0.001 for SIA_TCRP_). Furthermore, SIA_Post_ was significantly smaller than SIA_Ant_ and SIA_TCRP_ (all *p* < 0.001), while no significant difference was found between SIA_Ant_ and SIA_TCRP_ in both Group T and Group S (*p* = 1.000 for Group T and *p* = 0.943 for Group S).

**Table 3:** Comparison of surgically induced astigmatism between temporal clear corneal incision group and superior clear corneal incision group (mean ± SD)

Incision	Anterior		Posterior		Total	
	Temporal	Superior	Temporal	Superior	Temporal	Superior
SIA	0.33 ± 0.36 @81	0.55 ± 0.41 @4	0.09 ± 0.10 @174	0.13 ± 0.11 @96	0.28 ± 0.41 @83	0.50 ± 0.44 @3
p	< 0.001*		0.006*		0.001*	

SD: standard deviation; SIA: surgically induced astigmatism

With Bonferroni correction, a p value of < 0.017 was considered statistically significant

* Significant difference between Group T and Group S

**Fig. 2:**
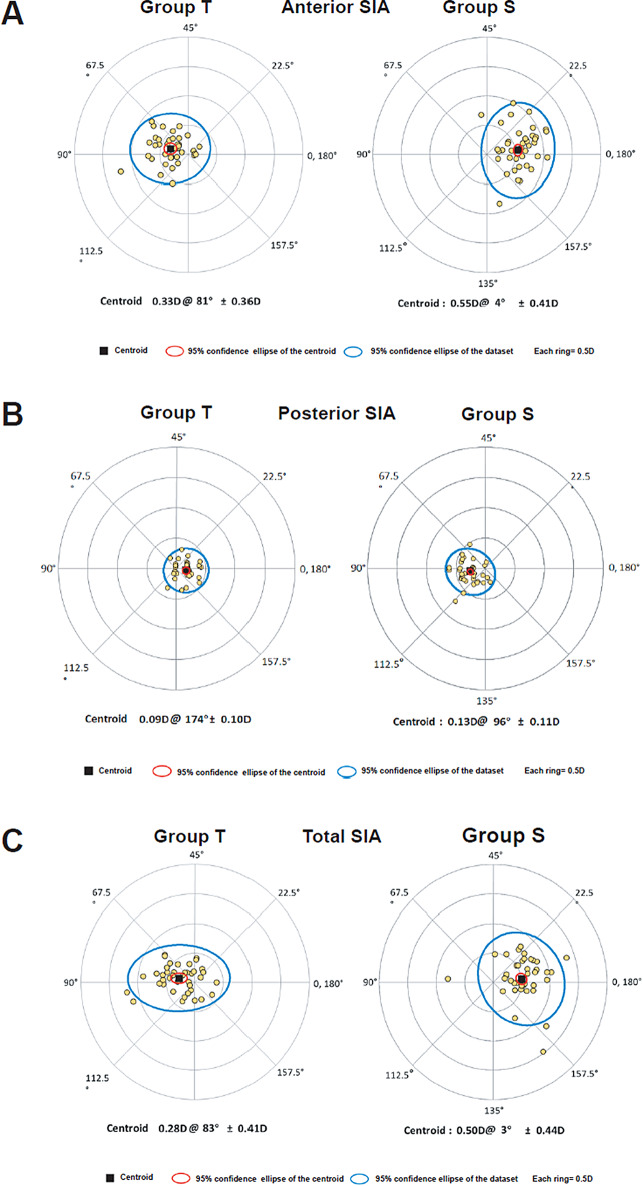
Double angle plots of the SIA distribution of the anterior (**A**), posterior (**B**), and total (**C**) cornea 3 months after ICL implantation with a temporal clear corneal incision and a superior clear corneal incision. SIA: surgical induced astigmatism

Double angle plots involving individual SIA distribution of the anterior (SIA_Ant_), posterior (SIA_Post_), and total (SIA_TCRP_) cornea are presented in Fig. 2.

## Discussion

Astigmatism is well known as a major cause of ocular refractive defects requiring optical correction. Minimizing astigmatism is important for achieving good uncorrected visual acuity postoperatively. Although the SIA as a result of corneal incision is small, it should not be neglected when determining the parameters of implanted ICL preoperatively because the objective of ICL implantation is to eliminate both spherical and cylindrical errors. Thus, evaluating and controlling SIA during ICL implantation would assist in eliminating postoperative astigmatism, increasing uncorrected visual acuity, and improving patient satisfaction.

Previously, Kamiya demonstrated that ICL implantation induced SIA by 0.48 ± 0.30 D and 0.57 ± 0.30 D through temporal and superior corneal incisions of 3.0 mm, respectively, by evaluating only the changes in the anterior cornea using an automated keratometer [9, 10]. Keratometry was previously used widely as an effective approach for evaluating corneal astigmatic changes; however, it can only evaluate the anterior corneal radius, and it is difficult to obtain information about the posterior corneal shape [19, 20]. With the development of the Pentacam (computer-assisted video keratography), a comprehensive and objective assessment of corneal tomography and topography including anterior, posterior, and total cornea could be realized in optometry and ophthalmology [21]. Previous studies have explored clear corneal incision-induced SIA after cataract surgery by using the Pentacam. Most studies have shown that the shape change and SIA of the total cornea were not proportional to those of the anterior cornea. The reports assumed that changes in the posterior cornea could not be ignored because the alterations were significant. They assumed that not only anterior corneal astigmatism but also posterior corneal astigmatism may have an obvious influence on decreasing postoperative refractive error after cataract surgery [17, 22]. Moreover, taking posterior corneal curvature into account when carrying out refractive lens exchange (RLE) surgery could decrease systematic measurement errors and potentially improve postoperative refractive predictability. However, Klijn obtained conflicting results in cataract surgery. They demonstrated that SIA of the posterior corneal surface is not clinically significant and could be neglected [23]. Therefore, we consider that assessing the shape and astigmatism of not only the anterior cornea, but also the posterior and total cornea, may be essential for evaluating the actual change in corneal shape and astigmatism after implanting ICL more precisely.

In the present study, we found that temporal incisions create astigmatism with a steep axis toward 90º, while the superior incision with a steep axis tends toward 180º in the anterior cornea, which is consistent with the results after cataract surgery [17, 24–26]. Furthermore, the change in total cornea astigmatism was similar to that in the anterior cornea, because no significant differences were found for SIA between the anterior and total cornea. However, clear cornea incision-induced shape change was somewhat different on the posterior surface, with the SIA 0.09 ± 0.10 D at a meridian of 174º with temporal incision and 0.13 ± 0.11 D at a meridian of 95º with superior incision, respectively. It indicated that, contrary to anterior and total cornea, temporal incisions create astigmatism with a steep axis toward 180º, while superior incision with steep axis tends toward 90º in the posterior cornea. Consistent with our results, Hayashi reported that horizontal corneal incision induced different SIA in the total and posterior cornea, with a WTR shift in the total cornea and an against-the-rule (ATR) shift in the posterior cornea [17, 27]. However, the SIA of temporal corneal incisions in Hayashi’s reports were higher than those in the present study. It may because cataract surgeries need more operation time and manipulation which would cause more severe cornea edema. Besides, the final follow up time were 8 weeks in Hayashi’s reports, which were shorter than the 3 months in the present study [17, 27]. Although the orientation of posterior cornea SIA is different from those of anterior and total cornea, the magnitude of the posterior SIA was small, and there was no statistical difference between SIA_Ant_ and SIA_Post_. As a result, the surgically induced astigmatic change of the posterior corneal surface after ICL implantation is not clinically significant. As far as we know, no previous study has been reported to date on the SIA of the anterior, posterior, and total cornea in ICL-implanted eyes with a 3.0-mm corneal incision.

Consistent with Kamiya’s results [9, 10], we found that the magnitude of CA_Ant_ and CA_TCRP_ significantly increased with the temporal incision group, but significantly decreased with the superior incision 3 months postoperatively. This may be because superior incisions would create ATR astigmatism, and temporal incisions always induce WTR astigmatism in total and anterior cornea, while most of the patients in this present study had WTR astigmatism preoperatively. The increased magnitude of CA_Ant_ and CA_TCRP_ in the temporal incision group and the decreased magnitude of CA_Ant_ and CA_TCRP_ in the superior incision group could explain why CA_Ant_ and CA_TCRP_ was significantly higher in the temporal incision group 3 months postoperatively, as compared to those values in the superior incision group. Thus, according to the astigmatism change in the anterior and total cornea, we assumed that a superior incision may be suitable for correcting WTR astigmatism using a temporal incision to correct ATR astigmatism when implanting a non-toric ICL.

Furthermore, all of the SIA of the anterior, posterior, and total cornea with temporal cornea incisions were significantly lower than those with superior cornea incisions, which were consistent with previous results of ICL implantation and cataract surgery [26, 28–30]. As we all know, the horizontal cornea diameter is probably 1 mm wider than the vertical orientation. Therefore, a smaller SIA induced by the temporal incision revealed in our study may be due to the longer distance from the corneal center to the temporal incision site as compared with the superior incision, which makes the effect of a 3.0-mm temporal incision significantly weaker than that with a 3.0-mm superior incision. Moreover, the presence and blinking of the upper eyelid may induce wound distortion and cornea stretch in the superior incision during wound healing postoperatively. This may be one of the major reasons why the superior incision has more effect on corneal astigmatism change compared to the temporal incision. Because of the low impact on corneal astigmatism, we assumed that a temporal incision may be the best solution if there was little astigmatism before surgery or if the preoperative astigmatism would be corrected with toric ICLs.

One limitation of our study is that we recruited a relatively small sample. Moreover, the follow-up time was short at 3 months. Hence, further evaluation using a larger number of patients and a longer observation time is advisable. In addition, we did not compare the astigmatic results and visual acuity of the ICL implanted eye after surgery between the two kinds of corneal incision. As a result, it is still questionable whether SIA has a significant influence on the astigmatic outcomes of ICL implantation. Therefore, further study on the impact of SIA on clinical astigmatic outcomes of ICL implantation is required.

## Conclusion

In the present study, neither temporal nor superior corneal incision significantly affected the refractive power of the anterior, posterior, and total cornea after ICL implantation. However, temporal incisions create astigmatism with a steep axis toward 90º, while the superior incision with a steep axis tends towards 180º.

Therefore, we assumed that a superior incision may be suitable for correcting WTR astigmatism, while a temporal incision is best for correcting ATR astigmatism when using a non-toric ICL. Meanwhile, the SIA of all the anterior, posterior, and total cornea were significantly higher with the superior incision as compared to the temporal incision. Thus, temporal incision could be a better choice with little preoperative astigmatism, or the preoperative astigmatism would be corrected with toric ICLs.

## Data Availability

The datasets used and/or analysed during the current study are available from the corresponding author on reasonable request.

## Abbreviations

ICL: implantable collamer lens
ECD: endothelium cell density
SD: standard deviation
D: diopters
SE: Spherical Equivalent
IOP: intraocular pressure
CCT: central corneal thickness
PCT: peripheral corneal thickness
Sim-K_Ant_: anterior simulated keratometry
CA_ant_: anterior corneal curvature astigmatism
Sim-K_Post_: posterior simulated keratometry
CA_post_: posterior corneal curvature astigmatism
TCRP: total corneal refractive power
CA_TCRP_: total corneal refractive power astigmatism
SIA: surgical induced astigmatism
SIA_Ant_: SIA of anterior cornea
SIA_Post_: SIA of posterior cornea
SIA_TCRP_: SIA of total cornea
